# A case of emphysematous cystitis: Timely recognition is the key

**DOI:** 10.4103/0971-4065.78084

**Published:** 2011

**Authors:** N. Relia, K. Chhavi

**Affiliations:** Department of Internal Medicine, University of Arkansas for Medical Sciences, Little Rock, AR, USA; 1 Departemnt of Radiology, University of Arkansas for Medical Sciences, Little Rock, AR, USA

A 58-year-old male presented to the Emergency Department with a 1-day history of fever, dysuria, and pain lower abdomen. Past medical history was significant for DM type 2 for which patient was on oral hypoglycemic agents. Patient was slightly hypotensive on arrival (BP 90/60 mmHg) and tachycardic (HR-127). Physical examination was remarkable for marked suprapubic tenderness. Laboratory analysis showed an elevated white count of 18000/μl, glucose of 400 mg/dl and creatinine of 1.7 mg/dl. Urinalysis revealed significant bacteriuria (3+) and ~800 wbc/hpf, ketones were, however, negative. Most recent HbA1C was 9.8% reflecting a poor glycemic control. Patient was aggressively resuscitated with IV fluids, switched to subcutaneous regular insulin for glycemic control, and initiated empirically on IV piperacillin/tazobactam. Patient, however, continued to complain of lower abdominal pain despite adequate urine output. A plain radiograph of the abdomen [ Figure 1a] obtained at day 2 showed extensive radiolucent collection in the bladder wall suggestive of air. Computed tomography of the abdomen/pelvis obtained same day revealed multiple linear lucencies with in the bladder wall surrounding the contrast-filled lumen [Figure 1b]. There was no air in the renal parenchyma, pelvis or renal vasculature. By day 3 (48 hrs of antibiotics) patient started showing improvement in his symptoms and urine culture grew Klebsiella pneumonia. The susceptibilities became available a day later and K. pneumonia was pan sensitive to multiple antibiotics including piperacillin/tazobactam. The antibiotics were continued for 7-days. Patient continued to improve and a plain radiograph of abdomen obtained on day 6 [Figure 2] showed complete resolution of air within the bladder wall.

**Figure 1:**
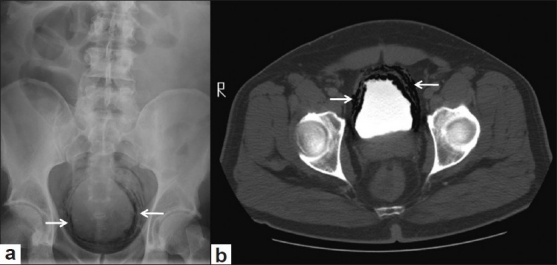
(a) Plain radiograph of the abdomen showing curvilinear lucencies in the region of urinary bladder in pelvis. (b) Axial CT section through pelvis confirms location of air within the bladder wall surrounding the contrast filled lumen

**Figure 2:**
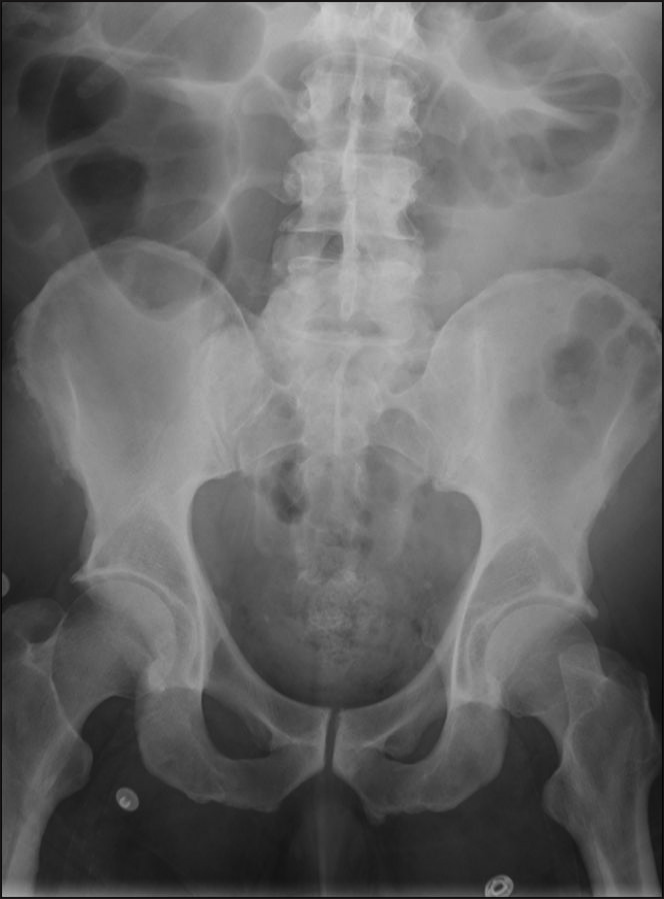
Follow-up radiograph of abdomen showing complete disappearance of air following treatment

## Discussion

Emphysematous cystitis is a rare disease responsible for a very small percentage of bacterial infections of the urinary tracts.[1] The infection can be rapidly progressive and fatal leaving a narrow window of opportunity for timely intervention of medical management. Elderly females with diabetes mellitus and patients with neurogenic bladder, obstructive uropathy, and recurrent urinary tract infections are among the most susceptible.[2] Infections are commonly due to *Escherichia coli, Klebsiella* species, or mixed infections and infrequently due to *Proteus, Citrobacter, Streptococci*, and *Candida*.[1] Treatment includes timely recognition and institution of parenteral antimicrobial agents without which it can progress rapidly to involve the upper urinary tract and vessels with death rates approaching as high as 70%–90%.[3]

Our case emphasizes the fact that early detection and appropriate treatment result in a favorable prognosis and that clinicians should maintain a high index of suspicion of this rare life-threatening complication especially in an elderly diabetic population presenting with urinary tract infection.
